# Metformin enhances tamoxifen-mediated tumor growth inhibition in ER-positive breast carcinoma

**DOI:** 10.1186/1471-2407-14-172

**Published:** 2014-03-11

**Authors:** Ji Ma, Yan Guo, Suning Chen, Cuiping Zhong, Yan Xue, Yuan Zhang, Xiaofeng Lai, Yifang Wei, Shentong Yu, Jian Zhang, Wenchao Liu

**Affiliations:** 1 The State Key Laboratory of Cancer Biology and Department of Oncology, Xijing Hospital, The Fourth Military Medical University, Xi’an 710032, China; 2 The State Key Laboratory of Cancer Biology and Department of Biochemistry and Molecular Biology, Xijing Hospital, The Fourth Military Medical University, Xi’an 710032, China; 3 Department of Breast Surgery, Lanzhou General Hospital of PLA, Lanzhou 730000, China; 4 Department of Pharmacy, Xijing Hospital, The Fourth Military Medical University, Xi’an 710032, China; 5 Department of Ear Nose Throat Surgery, Lanzhou General Hospital of PLA, Lanzhou 730000, China

**Keywords:** Metformin, Tamoxifen, Estrogen receptor, Breast cancer

## Abstract

**Background:**

Tamoxifen, an endocrine therapy drug used to treat breast cancer, is designed to interrupt estrogen signaling by blocking the estrogen receptor (ER). However, many ER-positive patients are low reactive or resistant to tamoxifen. Metformin is a widely used anti-diabetic drug with noteworthy anti-cancer effects. We investigated whether metformin has the additive effects with tamoxifen in ER-positive breast cancer therapy.

**Methods:**

The efficacy of metformin alone and in combination with tamoxifen against ER-positive breast cancer was analyzed by cell survival, DNA replication activity, plate colony formation, soft-agar, flow cytometry, immunohistochemistry, and nude mice model assays. The involved signaling pathways were detected by western blot assay.

**Results:**

When metformin was combined with tamoxifen, the concentration of tamoxifen required for growth inhibition was substantially reduced. Moreover, metformin enhanced tamoxifen-mediated inhibition of proliferation, DNA replication activity, colony formation, soft-agar colony formation, and induction of apoptosis in ER-positive breast cancer cells. In addition, these tamoxifen-induced effects that were enhanced by metformin may be involved in the bax/bcl-2 apoptotic pathway and the AMPK/mTOR/p70S6 growth pathway. Finally, two-drug combination therapy significantly inhibited tumor growth in vivo.

**Conclusion:**

The present work shows that metformin and tamoxifen additively inhibited the growth and augmented the apoptosis of ER-positive breast cancer cells. It provides leads for future research on this drug combination for the treatment of ER-positive breast cancer.

## Background

Tamoxifen, a non-steroidal anti-estrogen, is the most widely used anti-estrogen for the treatment or prevention of estrogen receptor (ER)-positive breast cancer [1,2]. For women with ER-positive breast cancer, treatment for 5 years with adjuvant tamoxifen substantially reduces the rate of recurrence [3]. Recent trials have shown that continuing tamoxifen for 10 years rather than stopping at 5 years produces a further reduction in recurrence and mortality [4]. However, many ER positive patients are low reactive or resistant to tamoxifen [3] and such long treatment with tamoxifen causes serious side-effects such as increases in endometrial hyperplasia and carcinomas [5], an excess of venothrombotic episodes, and the development of de novo or acquired tamoxifen resistance [6,7]. Thus, there is the need for a more effective therapy with fewer side-effects for these patients. Modulation of tamoxifen sensitivity that results in lessening of its side-effects is a desirable goal.

Metformin (1,1-dimethylbiguanide hydrochloride) is a biguanide commonly used to treat type 2 diabetes mellitus. It is frequently referred to as an “insulin sensitizer” because it lowers circulating insulin levels in settings of insulin resistance and hyperinsulinemia [8,9]. Much recent interest has focused on the antitumor effects of metformin. A recent retrospective analysis examined the effects of metformin on potentiation of chemotherapy in breast cancer patients and found that women with diabetes and breast cancer receiving metformin and neoadjuvant chemotherapy experienced a higher pathologic complete response rate than diabetic patients just receiving neoadjuvant chemotherapy, but not metformin [10]. This study generated substantial enthusiasm that metformin might enhance the efficacy of other anti-tumor agents, particularly endocrine drugs. In addition, emerging evidence suggests that metformin may activate AMPK and inhibit mTOR signaling to exert anti-tumor effects [11-13]. Of note, tamoxifen also represses mTOR signaling to restrain ER-positive breast cancer growth [14]. In a phase II GINECO study, everolimus, an mTOR inhibitor, also enhanced the efficacy of tamoxifen in ER-positive metastatic breast cancer patients [15]. It was postulated that the pleiotropic actions of metformin on AMPK/mTOR signaling might play an additive role with tamoxifen and allow its use in non-diabetic patients and women with ER-positive tumors.

Based on these considerations, we posed the following questions: first, whether metformin and tamoxifen have synergic effects on the growth of ER-positive breast cancer cells; second, whether AMPK/mTOR or other signaling pathways are involved in the two-agent synergic effects; and last, whether this synergic effect occurs in vivo. In this study, we tested the hypothesis that the combination of metformin and tamoxifen suppresses the growth of ER-positive breast cancer, and we provide evidence of basic research for future clinical trials.

## Methods

### Reagents and antibodies

Metformin (1,1-dimethylbiguanide hydrochloride), tamoxifen (4-hydroxytamoxifen), thiazolyl blue tetrazolium bromide (MTT), Giemsa stain, dimethyl sulfoxide (DMSO) and agarose were purchased from Sigma-Aldrich (St. Louis, MO, USA). Metformin was dissolved in sterile water to make a 1 M stock solution, and tamoxifen was dissolved in ethanol to make a 3.2 mM stock solution. The Cell-LightTM BrdU DNA Cell Proliferation Kit was purchased from Ribobio (Guangzhou, China). The antibodies against p-AMPK (Thr172), AMPK, p-mTOR (Ser2448), mTOR, p-p70S6 (Thr389), p70S6, bcl-2 and bax are rabbit monoclonals and were purchased from Cell Signaling Technologies (Beverly, MA, USA). The antibody against β-actin is a mouse monoclonal and was purchased from Boster (Wuhan, China).

### Cell culture

The human breast cancer cell lines MCF-7 and ZR-75-1 were obtained from the American Type Culture Collection. The cells were cultured in DMEM supplemented with 10% fetal bovine serum (FBS) and were maintained in a humidified environment containing 5% CO_2_ and air at 37°C. The culture medium of MCF-7 cells contained 0.01 mg/ml insulin.

### Cell survival assay

Cells were seeded in a 96-well plate (5 × 10^3^ cells per well) and incubated for 24 h. After treatment with different drugs for 48 or 96 h, the cells were then incubated with 0.5 mg/ml MTT (Sigma). Four hours later, the medium was replaced with 150 μl dimethyl sulfoxide (DMSO) (Sigma) and vortexed for 10 min. Absorbance was then recorded at 490 nm using an Infinite® F500 micro-plate reader (TECAN). Relative values of optical density were calculated.

### DNA replication activity assay

DNA replication activity was examined using BrdU (5-bromo-2-deoxyuridine). Cells grown on coverslips (Fisher) were treated with different drugs for 48 h. The cells were then incubated with BrdU for 1 h and stained with an anti-BrdU antibody (Ribobio) according to the manufacturer’s instructions. The results were analyzed using a fluorescence microscope (Olympus).

### Plate colony formation assay

Cells (1 × 10^3^) treated with different drugs were seeded into 60 mm dishes with 5 ml of DMEM. After 10 days, the resulting colonies were rinsed with PBS, fixed with methanol at -4°C for 5 min, and stained with Giemsa (Sigma) for 20 min. Counting was performed only on clearly visible colonies (diameter > 50 μm).

### Soft-agar assay

Cells (1 × 10^3^) were added to 3 ml of DMEM with 0.3% agar and layered onto 6 ml of 0.5% agar beds in 60 mm dishes. Cells were treated with different drugs and cultured for 2 weeks, after which colonies were photographed. Colonies larger than 50 μm in diameter were counted as positive for growth.

### Flow cytometry analysis

Cells (5 × 10^5^) were collected and washed twice with PBS and then resuspended in 500 μl of staining solution containing fluorescein isothiocyanate (FITC)-conjugated annexin V antibody (5 μl) and propidium iodide (PI, 5 μl of 250 μg/ml stock solution). After incubation for 15 min at room temperature in the dark, cells were immediately analyzed on a flow cytometer. Apoptotic cells were double stained with annexin V and PI. The percentage of cells undergoing apoptosis was determined.

### Western blot

Western blot was performed as described previously [16]. The blots were probed with the different primary antibodies and species-matched secondary antibodies. The bands were detected using enhanced chemiluminescence (Pierce) or the Odyssey Imaging System (LiCor Biosciences).

### Xenograft study in nude mice

Three days after injection of estrogen in the abdomen (E2, 0.9 mg/kg, every three days), 5 × 10^6^ MCF-7 cells were injected into the abdominal mammary fat pad of 4-week-old female nude mice. When tumor volume reached approximately 200 mm^3^, we randomly allocated the mice to groups in which they received PBS, metformin, tamoxifen, or a combination of the two drugs. Tumor growth was monitored by caliper measurements. Excised tumors were weighed, and portions were frozen in liquid nitrogen or fixed in 4% paraformaldehyde for further study.

### Animal ethics statement

This study was performed in strict accordance with the recommendations in the Guide for the Care and Use of Laboratory Animals of the Forth Military Medical University. The protocol was approved by the Committee on the Ethics of Animal Experiments of the Forth Military Medical University. All surgery was performed under sodium pentobarbital anesthesia, and every effort was made to minimize suffering.

### Immunohistochemistry

Immunohistochemical staining was performed as described previously [16] using rabbit anti-Ki67, anti-p-AMPK, anti-p-mTOR, and anti-p-p70S6 from Cell Signaling (Beverly, MA, USA) as primary antibodies.

### Statistical analysis

Data from all quantitative assays are expressed as the mean ± standard deviation and were analyzed statistically using a one-way analysis of variance (ANOVA) and the independent-samples *t* test. Statistical calculations were performed using SPSS 14.0. *P* values of less than 0.05 were considered statistically significant.

## Results

### Inhibition of the viability of ER-positive breast cancer cells by tamoxifen plus metformin

**Figure 1:**
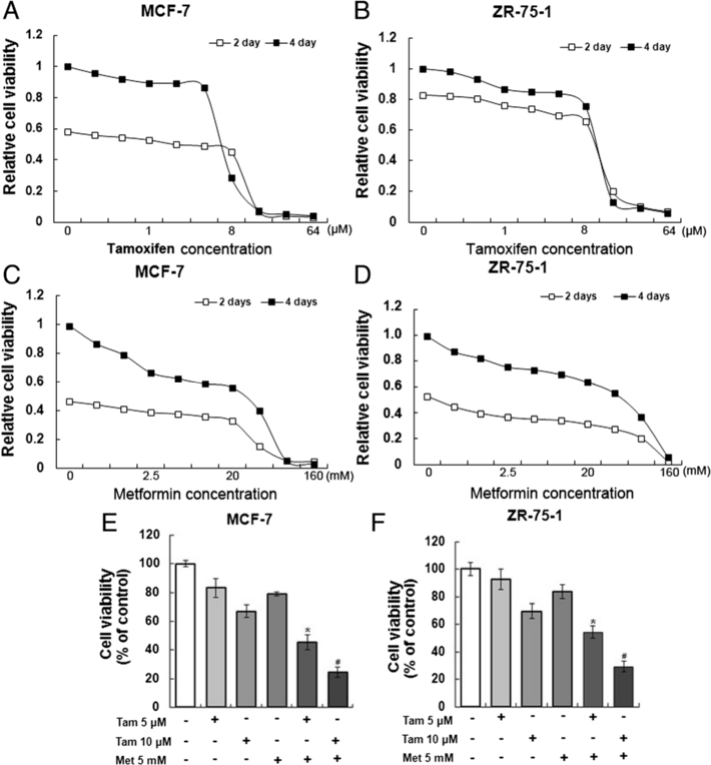
**Metformin and tamoxifen inhibit the viability of MCF-7 and ZR-75-1 cells. (A-B)** MCF-7 and ZR-75-1 cells were treated with tamoxifen at different concentrations (0, 0.25, 0.5, 1, 2, 4, 8, 16, 32 and 64 μM). After 2 or 4 days, the cell viability was measured. **(C-D)** MCF-7 and ZR-75-1 cells were treated with metformin at different concentrations (0, 0.625, 1.25, 2.5, 5, 10, 20, 40, 80 and 160 mM). After 2 or 4 days, the cell viability was measured. **(E-F)** MCF-7 and ZR-75-1 cells were treated with 5 or 10 μM tamoxifen, 5 mM metformin, or a combination of the two agents for 4 days, and the cell viability was measured. Histograms represent quantification of cell viability. All data are expressed as the mean ± standard deviation (SD) from three independent experiments. *P < 0.05 for the 5 μM Tam group vs. the 5 μM Tam + 5 mM Met group; ^#^*P* < 0.05 for the 10 μM Tam group vs. the 10 μM Tam + 5 mM Met group. Tam and Met represent tamoxifen and metformin, respectively.

To assess the effect of combining tamoxifen with metformin on the viability of ER-positive breast cancer cells, MCF-7 and ZR-75-1 cells were first treated with tamoxifen or metformin individually. Cell proliferation was measured at 2 and 4 days after treatment. The inhibitory effects of both tamoxifen and metformin on the two cell lines were significantly dose dependent and at the concentration of about 8 μM tamoxifen or 20 mM metformin the cell survival curves began to drop (Figure 1A-D). To avoid the inhibitory effect of high concentration metformin on breast cancer cells, we just used a low concentration metformin (5 mM) and we combined 5 mM metformin with a low concentration tamoxifen (5 μM) or a high concentration tamoxifen (10 μM) to investigate the additive effect of two drugs. The results showed that the low concentrations of metformin and tamoxifen additively inhibited MCF-7 cell proliferation compared with the single tamoxifen treatment (Figure 1E). Similar results were found using the ZR-75-1 cell line (Figure 1F).

### Inhibition of the DNA replication activity of ER-positive breast cancer cells by tamoxifen plus metformin

**Figure 2:**
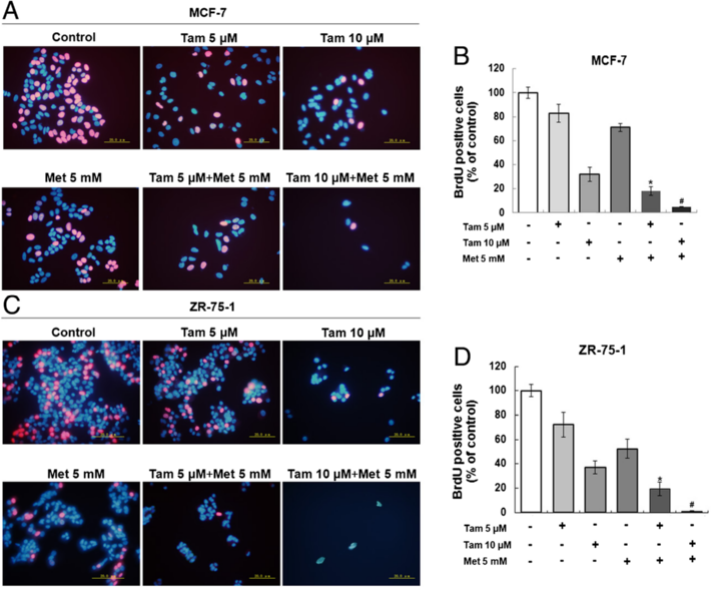
**Metformin and tamoxifen inhibit the DNA replication activity of MCF-7 and ZR-75-1 cells. (A and C)** MCF-7 and ZR-75-1 cells were treated with 5 or 10 μM Tam, 5 mM Met, or 5 or 10 μM Tam + 5 mM Met. After 2 days, the DNA replication activity of the cells was measured by BrdU assay. A red color indicates cells in the DNA replication phase, and a blue color indicates the cell nucleus (scale bar = 20.0 μm). **(B and D)** BrdU-positive cells were counted in five random high-power fields (original magnification, 400x). Histograms represent quantification of BrdU-positive cells. All data are expressed as the mean ± standard deviation (SD) for three independent experiments. *P < 0.05 for the 5 μM Tam group vs. the 5 μM Tam + 5 mM Met group; ^#^*P* < 0.05 for the 10 μM Tam group vs. the 10 μM Tam + 5 mM Met group.

DNA replication activity is a critical index for cell growth. BrdU, a synthetic thymidine analogue that binds to replicating DNA, was used to examine the rate of DNA replication. After 3 h incubation with BrdU, the cells in the DNA replication phase were labeled with a red color. As shown in Figure 2A and B, 5 mM metformin or 5 μM tamoxifen alone had little effect on MCF-7 DNA synthesis, but the combination of the two agents significantly inhibited DNA synthesis compared with the single-agent treatments. Moreover, the combination of 5 mM metformin with 10 μM tamoxifen had an even larger inhibitory effect on DNA replication. The ZR-75-1 cell line showed similar effects with the different treatment conditions (Figure 2C and D).

### Inhibition of colony formation of ER-positive breast cancer cells by tamoxifen plus metformin

**Figure 3:**
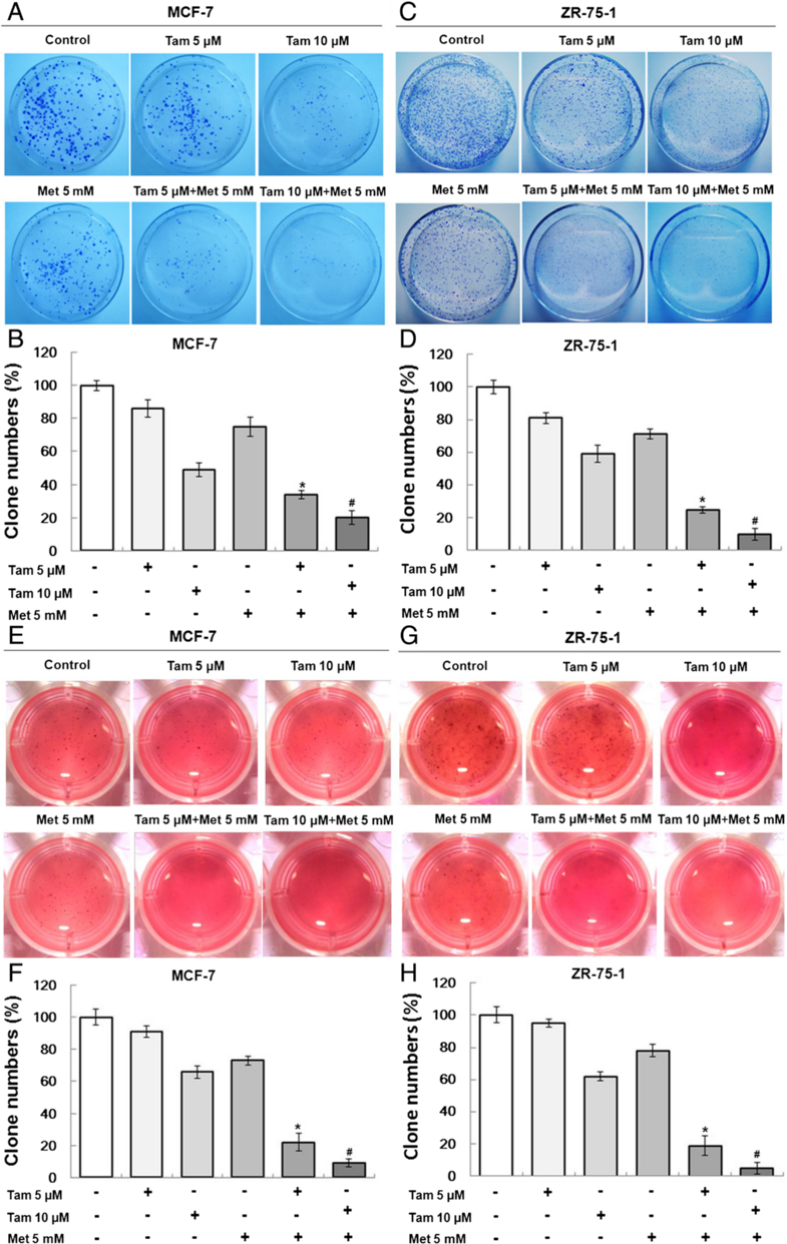
**Metformin and tamoxifen inhibit the colony-forming ability of MCF-7 and ZR-75-1 cells.** The colony formation assays were performed as described in the “Methods”. **(A, C, E and G)** MCF-7 and ZR-75-1 cells were treated with 5 or 10 μM Tam, 5 mM Met, or 5, or 10 μM Tam + 5 mM Met. Typical cell colonies were imaged and are shown. **(B, D, F and H)** The number of colonies formed was counted for different groups. Histograms represent the quantification of the number of colonies formed. All data are expressed as the mean ± standard deviation (SD) for three independent experiments. *P < 0.05 for the 5 μM Tam group vs. the 5 μM Tam + 5 mM Met group; ^#^*P* < 0.05 for the 10 μM Tam group vs. the 10 μM Tam + 5 mM Met group.

The colony formation of tumor cells represents the degree of malignancy and tumorigenicity. We examined the effect of tamoxifen plus metformin on the ability of cells to form colonies using plate and soft-agar colony formation assays. In long-term (10 or 14 days) clonogenicity assays, 5 μM and 10 μM tamoxifen had weak inhibitory effects on the colony formation of MCF-7 and ZR-75-1 cells. However, after the addition of 5 mM metformin to the tamoxifen, the number of colonies formed by the cells was reduced significantly (Figure 3A-D). Similar inhibitory effects of the two agents were found using a soft-agar colony formation assay (Figure 3E-H).

### Promotion of apoptosis of ER-positive breast cancer cells by tamoxifen plus metformin

**Figure 4:**
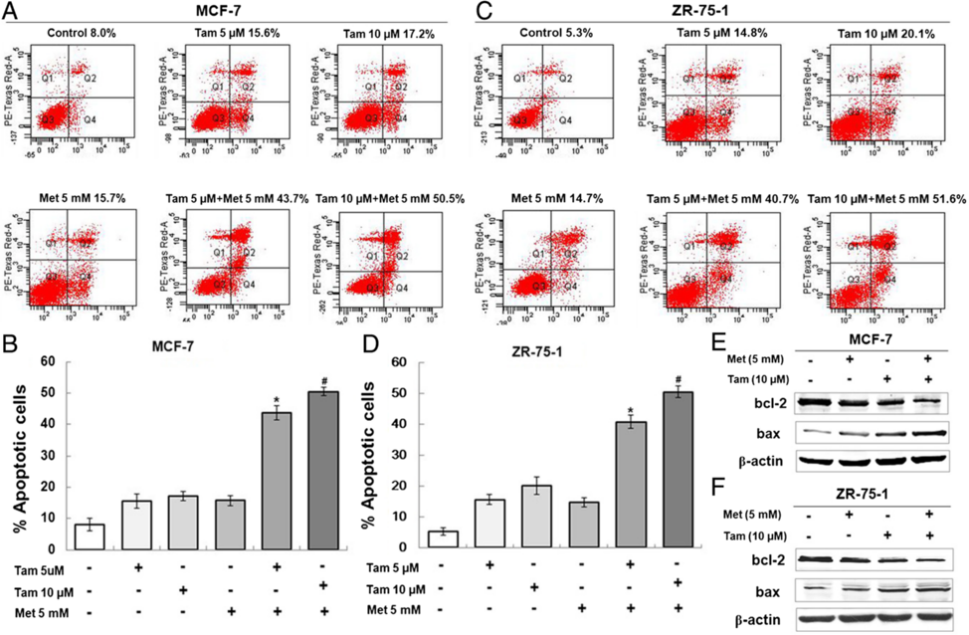
**Metformin and tamoxifen promote the apoptosis of MCF-7 and ZR-75-1 cells.** MCF-7 and ZR-75-1 cells were treated with 5 or 10 μM Tam, 5 mM Met, or 5 or 10 μM Tam + 5 mM Met. After 4 days, apoptosis was measured. **(A and C)** Annexin/propidium iodide staining was used to examine apoptosis of the cells. **(B and D)** Histograms represent the quantification of apoptotic cells. *P < 0.05 for the 5 μM Tam group vs. the 5 μM Tam + 5 mM Met group; ^#^*P* < 0.05 for the 10 μM Tam group vs. the 10 μM Tam + 5 mM Met group. **(E and F)** MCF-7 and ZR-75-1 cells were treated with 5 mM Met, 10 μM Tam or 10 μM Tam + 5 mM Met for 4 days. Protein was extracted and subjected to western blot. The expression of the target proteins was normalized to β-actin.

Because the combination of tamoxifen plus metformin caused a significant reduction in the growth of ER-positive breast cancer cells, the underlying mechanisms were investigated. First, we measured the induction of apoptosis by this combination. Cells were treated with the agents individually or in combination and examined by annexin V/propidium iodide staining (Figure 4A-D). At the concentrations tested, single treatments of 5 μM or 10 μM tamoxifen or 5 mM metformin produced a minor rate of apoptosis, but the combination induced apoptosis in almost 50% of the cells. To further examine the processes of cell death induced by this combination, we then analyzed cell extracts for the expression of biological markers of apoptosis. Western blot results showed that the combination drug treatment resulted in significant induction of the pro-apoptotic gene bax, whereas the expression of the anti-apoptotic gene bcl-2 was decreased (Figure 4E-F).

### Activation of AMPK and inhibition of mTOR/p70S6 signaling by tamoxifen plus metformin

**Figure 5:**
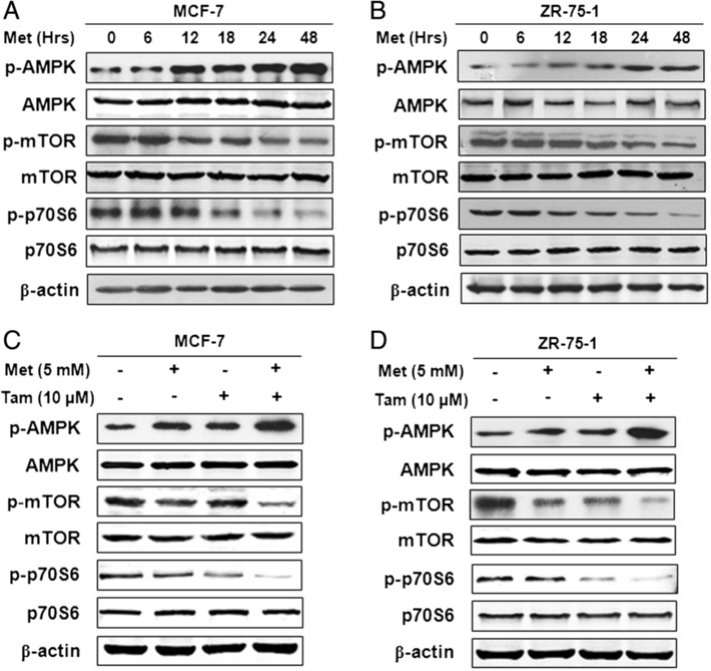
**Detection of the AMPK/mTOR signaling pathway after combination treatment with metformin and tamoxifen in MCF-7 and ZR-75-1 cells. (A-B)** MCF-7 and ZR-75-1 cells were treated with 5 mM Met for different times (0, 6, 12, 18, 24 and 48 hours). **(C-D)** MCF-7 and ZR-75-1 cells were treated with 5 mM Met, 10 μM Tam or 10 μM Tam + 5 mM Met for 2 days. Protein was extracted and subjected to western blot. The expression of the target proteins was normalized to β-actin.

To investigate the mechanisms by which tamoxifen plus metformin induced growth inhibition, we first evaluated the effects of metformin alone and found that metformin increased the levels of activated AMPK (p-AMPK, with no change in AMPK levels) in a time-dependent manner in MCF-7 and ZR-75-1 cells (Figure 5A and B). The phosphorylation of the signaling molecules downstream of mTOR and p70S6, was significantly decreased following treatment, yet the total levels of mTOR and p70S6 were minimally affected (Figure 5A and B). We then examined the effects of the combination of metformin and tamoxifen on these signals. As shown in Figure 5C and 5D, the combination of these two agents caused an increase in phosphor-AMPK and a reduction in phosphor-mTOR and phosphor-p70S6, but total AMPK, mTOR and p70S6 levels were unchanged.

### Metformin plus tamoxifen inhibit the growth of ER-positive breast cancer *in vivo*

**Figure 6:**
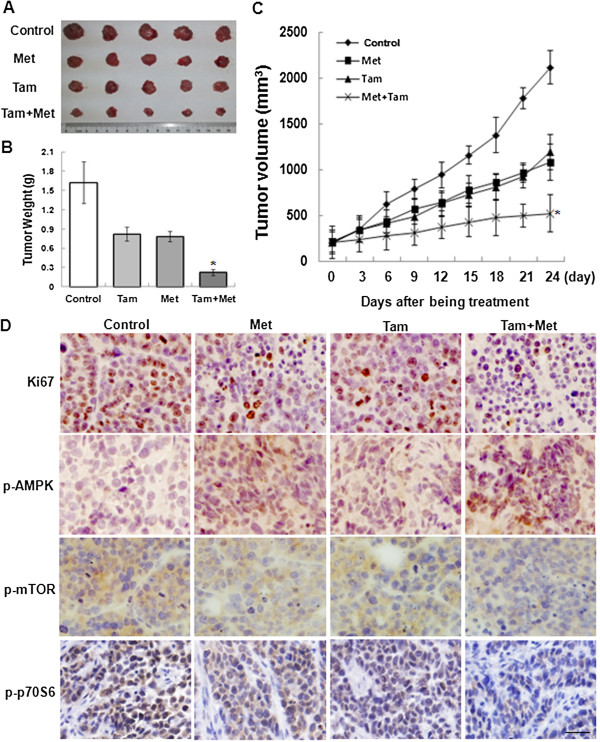
**Inhibitory effects of metformin and tamoxifen on tumor growth in a nude mouse model.** These experiments were performed as described in the “Methods”. When the tumors in nude mice reached 200 mm^3^, the mice were given metformin (2 mg/ml) in their drinking water, tamoxifen (60 mg/kg) through oral gavage or the two agents simultaneously. The tumor growth was assessed every 3 days until day 24 of treatment by measuring two perpendicular diameters and calculating the volume in mm^3^. **(A)** Photographs of typical tumors are shown. **(B)** Histograms represent the weight of tumors from the different groups. **(C)** Tumor growth curve. Statistical analysis was performed using one-way ANOVA and Student’s *t*-test day with the day 24 values only. *P < 0.05 compared with the Tam group. **(D)** Intratumoral target protein expression was assessed by Ki67, p-AMPK, p-mTOR and p-p70S6 immunolabeling on paraffin-embedded tumor sections. Representative images are shown (original magnification, 400x).

To further evaluate the effects of metformin alone and in combination with tamoxifen on tumor growth in vivo, MCF-7 cells were injected into nude mice. When the tumors reached approximately 200 mm^3^, the mice were then treated with metformin (2 mg/ml) in the animals’ drinking water, tamoxifen (60 mg/kg) by oral gavage, or the two agents simultaneously. As shown in Figure 6A and 6C, the combination (Met + Tam) group achieved a sustained and significant arrest of tumor growth equal to a 75.4% decrease in mean tumor volume on 24 day after being treatment compared with the control group. However, the groups that received metformin or tamoxifen alone exhibited tumor growth that was inhibited by 48.9% and 43.8%, respectively, on 24 day after being treatment compared with the control group. Moreover, the mice were sacrificed, and the tumor weights were examined. The tumor weight of the combination treatment group was the lightest of the four groups (Figure 6B). Finally, immunolabeling for Ki-67, p-AMPK, p-mTOR and p-p70S6 was analyzed. Consistent with results observed in cultured cells, these proteins were lower in the two groups receiving either metformin or tamoxifen compared with the control group. Importantly, combining the two agents significantly inhibited the expression of these proteins (Figure 6D).

## Discussion

Recently, the ATLAS clinical trial announced the latest results that continuing tamoxifen to 10 years rather than stopping at 5 years further reduces recurrence and mortality in patients with ER-positive breast cancer [4]. This is encouraging news for the efficacy of tamoxifen, although its significant problems, therapeutic side-effects and resistance, must still be addressed. Some epidemiological studies suggest that metformin may exert anti-tumor effects in diabetic patients with breast cancer [10,17]. Because of its minor side-effects and low toxicity, metformin may be a desirable drug for the prevention of breast cancers and the growth reduction of existing tumors in women [18-20]. However, the effects of metformin on endocrine therapies for ER-positive breast cancer patients are largely unknown. Based on this consideration, we investigated the efficacy of low-concentration doses of metformin alone or in combination with tamoxifen in a model of ER-positive breast cancer that reflects treatment strategies in common clinical use. In this study, we demonstrate that metformin enhanced the inhibitory effects of tamoxifen on the growth of ER-positive breast cancer in vitro and in vivo. Notably, when metformin was combined with tamoxifen, the concentration of tamoxifen required to inhibit growth was substantially reduced. Our observations indicate the potential importance of such combined approaches for increasing tamoxifen efficacy or lessening its side-effects in the clinical setting.

Metformin is widely used for the treatment of diabetes mellitus type 2, where it reduces insulin resistance and diabetes-related morbidity and mortality [8,9]. Population-based studies show that metformin treatment is associated with a dose-dependent reduction in cancer risk [21,22]. Metformin treatment also increases complete pathological tumor response rates following neoadjuvant chemotherapy for breast cancer, suggesting a potential role as an anti-cancer drug [23-25]. Diabetes mellitus type 2 is associated with insulin resistance, elevated insulin levels and an increased risk of cancer and cancer-related mortality [26,27]. This increased risk may be explained by the activation of the insulin and insulin-like growth factor (IGF) signaling pathways and increased signaling through the estrogen receptor [28,29]. Reversal of these processes through reduction of insulin resistance by the oral anti-diabetic drug metformin is an attractive anti-cancer strategy. In this study, we also provide direct evidence that metformin acts additively with endocrine therapy for ER-positive breast cancer patients, who account for almost 70% of all breast cancers. Metformin enhanced the inhibitory effects of tamoxifen on proliferation, DNA replication activity, colony formation, and soft-agar colony formation in ER-positive breast cancer cells. An increasing number of studies suggest that metformin is an activator of AMPK, which inhibits protein synthesis and gluconeogenesis during cellular stress [12-14]. The main downstream effect of AMPK activation is the inhibition of mTOR signaling pathways [13,14]. Here we showed that in a time-dependent manner, metformin increased p-AMPK and decreased p-mTOR expression. p70S6, one of the critical downstream effectors of the mTOR signaling pathway, was also inhibited.

We found that the specific clinical circumstances in which metformin may be used in ER-positive breast cancer patients substantially influenced responsiveness to this agent. Our data are the first to examine the individual and combined effects of metformin and tamoxifen on a nude mouse model of ER-positive breast cancer and to investigate the apoptotic and growth pathways involved. Prior to this study, to our knowledge, there were two articles that described the effects of the combination of metformin and tamoxifen. One investigation suggested that metformin may decrease the density of endometrial glands and hyperplasia induced by tamoxifen in a mouse model [30]. The other team found that metformin and tamoxifen inhibited the proliferation of MCF-7 cells and that tamoxifen-resistant cells were less sensitive to tamoxifen than to metformin [31]. That study provided the important clue that metformin may exert an inhibitory effect on ER-positive breast cancer cells. It is consistent with our results that metformin and tamoxifen inhibit the growth of MCF-7 cells. The focus of our study was a wild-type, ER-positive breast cancer model, which represents patients receiving initial tamoxifen treatment. For these patients, it is possible that metformin additively augments tamoxifen efficacy but also suppresses tumor cell growth.

## Conclusion

Taken together, our data indicate that metformin plus tamoxifen may have synergic effects on ER-positive breast cancer cells and tumor growth. We have further confirmed that these synergic effects of the two agents may be involved in the bax/bcl-2 apoptotic pathway and the AMPK/mTOR/p70S6 growth pathway. Although the antitumor effect of metformin is complicated and not yet fully understood, our findings provide direct evidence of its efficacy on ER-positive breast cancer in combination with tamoxifen treatment.

## Competing interest

The authors declare no conflict of interest.

## Authors’ contributions

Designed the experiments: JM, JZ and WCL; Performed the experiments and analyzed data: JM, YG, SNC, STY and YZ; Wrote the manuscript: JM; Edited the manuscript: YX, CPZ, XFL and YFW. All authors read and approved the final manuscript.

## Pre-publication history

The pre-publication history for this paper can be accessed here:

http://www.biomedcentral.com/1471-2407/14/172/prepub
